# White spot lesions: diagnosis and treatment – a systematic review

**DOI:** 10.1186/s12903-023-03720-6

**Published:** 2024-01-09

**Authors:** Pedro C. Lopes, Teresa Carvalho, Ana T. P. C. Gomes, Nelio Veiga, Letícia Blanco, Maria José Correia, Anna Carolina Volpi Mello-Moura

**Affiliations:** 1 Faculty of Dental Medicine, Universidade Católica Portuguesa, Portugal PT, Centre for Interdisciplinary Research in Health (CIIS) - Universidade Católica Portuguesa, Viseu, PT Portugal; 2 Faculty of Dental Medicine, Universidade Católica Portuguesa, Lisbon, PT Portugal; 3 Department of Surgery, Universidad de Salamanca, Salamanca, Spain

**Keywords:** White spot lesion, Dental caries, Diagnosis, Treatment

## Abstract

**Background:**

White spot lesions represent the first stage of caries and their prevalence has been increasing in recent years, particularly in patients undergoing orthodontic treatment. DIferential diagnosis and lesion activity are essential to decide on the clinical approaches to treatment. The aim of this study is to understand if the new diagnostic tools such as fluorescence, microradiography and computed microtomography have the potential to change the conventional treatment of white spots”.

**Methods:**

A systematic search of available studies in the literature was carried out, using PRISMA guidelines, in Pubmed and Scopus electronic databases and manually to identify relevant articles to answer the PICO question: “Do the new diagnostic tools have the potential to change the conventional treatment of white spots?”. This systematic review included randomized controlled trials (RCT), cross-sectional and longitudinal studies complying with the following inclusion criteria: (i) studies in humans, (ii) studies about white spot lesions, (iii) studies published between 2012 and 2023, (iv) studies having both diagnosis and treatment and (v) studies with full text available. In this review we excluded other systematic reviews of clinical trials and in vitro studies. The RoB tool was used to assess the risk of bias.

**Results:**

The systematic literature search identified 143 potentially relevant references, which after applying the exclusion criteria, resulted in 20 articles. Regarding diagnostic methods, most articles found were based on conventional methods of visual examination (n:10) or fluorescence (n:7). The least referenced diagnostic techniques were based on the use of clinical photographs (n:2), cross-sectional microradiography (n:1) and computed microtomography (n:1). The use of DIAGNOdent was reported by 3 in vitro studies. With regard to therapies, most studies reported the use of infiltrating resin (n:7) and fluoride-based products (n:5). Other studies have reported the use of self-assembling peptide P11-4 (n:1), home care (n:1), casein phosphopeptide-amorphous calcium phosphate (n:2) and hydrochloric acid (n:1). Combination therapies were also considered.

**Conclusion:**

Diagnostic tool does not have the potential to change the form of treatment, whether it is a conventional method or a more differentiated one.

## Background

White spot lesions represent the first stage of dental caries and their prevalence has been increasing in recent years, corresponding to a value between 10 and 49% [1]. They frequently appear in patients undergoing orthodontic treatment, usually caused by the accumulation of bacterial plaque in the cervical region of the tooth [2]. Epidemiological indices are used to aid diagnosis, such as the Enamel Developmental Defects index (DDE index). This index allows the classification of lesions as demarcated, diffuse and hypoplastic. and histopathological changes are associated with its classification. The clinical aspects of discoloration are related to the optical properties described by the tooth itself. Value, hue and chroma are responsible for tooth color, however, its appearance is minutely affected by its translucency, opacity and fluorescence [2].

Clinically, the appearance of the lesion is opaque white, due to the optical phenomenon caused by mineral loss and the difference in the refractive index of water and air that fill the spaces formed in the enamel [1, 3, 4] In this way, the lesion will be *whitish* and with little translucency since there is an increase in enamel porosity. This surface irregularity then causes a loss of brightness, making the reflection of light diffuse [5, 6]. Regarding the time of formation, the lesions develop relatively quickly. However, they are only visible after the tooth surface has dried [5, 6]. Thus, the white spot is related to the loss of minerals by the enamel which, when diagnosed in its initial phase, is still partially demineralized and is subject to remineralization [7, 8].

White spot lesions are signs of demineralization under a layer of intact, highly mineralized enamel, which may or may not lead to the development of caries. Demineralization is a process that causes mineral loss from the enamel due to the dissolution of hydroxyapatite by the acid environment, thus creating porosities in the enamel [9]. A significant correlation can be found between the intensity of white spot lesion color and lesion volume. The intensity of the lesion color can predict the depth of enamel demineralization [9].

During orthodontic treatment, there is great difficulty in hygienic care for patients under certain conditions, particularly for those with fixed orthodontic appliances. Now, knowing that orthodontic appliances, due to their inherent characteristics, favor the retention and accumulation of bacterial plaque, hindering the normal cleaning process, this predicts a high predisposition to caries and associated periodontal problems [8]. One of the most frequent and undesirable sequelae after orthodontic treatment is the white spot lesion without cavitation caused by enamel demineralization [10]. Its formation is caused by several factors, among them we can mention poor oral hygiene, loose bands, dehydration of the tooth during cementation, and isolation of the insufficient area with a mixture of saliva and cement [11, 12]. Moreover, low salivary flow and low buffering capacity do not promote the reduction of microorganisms and food remains in the oral environment, which impairs the neutralization of acids and reduces the tendency to remineralization of the initial lesions of the tooth enamel, leading to caries development [13].

Traditionally, the diagnostic methods for white spot lesions are visual and photographic examination, in order to detect the depth and extent of the lesions. However, in recent years, new techniques have emerged such as fluorescence (the DIAGNOdent mechanism appears within fluorescence) and microradiography and microcomputed tomography. These techniques may be used in vivo *but* are currently used as research tools for in vitro studies,contributing to gaining knowledge, which may be translated to the clinical setting in the future. The aim is to make a more accurate diagnosis of the lesion, understanding it in terms of depth and extent. Therefore, the treatment will be more directed towards each particular injury, in order to improve the results and prognosis and, whenever possible, this treatment should be as conservative as possible.

With the evolution of Dentistry, there are now several treatments for white spot lesions, from more invasive methods to more conservative solutions, which try to preserve the dental structures as much as possible. Within this conservative concept various strategies can be used, such as oral hygiene instructions, prescription of topical fluorides, including fluoride varnish, use of casein phosphopeptide-amorphous calcium phosphate (CPP-ACP) and infiltrating resin [14].

There are several forms of diagnosis and suitable treatments for white spot lesions, due to different clinical situations. Knowing how to identify a white spot lesion and ensure correct and effective treatment is crucial to promote good care and achieve clinical success. The objective of this review is to clarify information on the diagnosis and treatment of this type of lesion, as it is much easier to diagnose cavitated lesions than white spot lesions and even evaluate the activity of these lesions. For that, we want to understand if the new diagnosis tools can change white spot’s conventional treatment, through a systematic review of the literature.

## Methods

To fulfil the proposed objective, a PICO(S) question was formulated: “Do the new diagnostic tools have the potential to change the conventional treatment of white spots?”. A systematic search of available studies in literature was conducted in the electronic databases Pubmed and Scopus to identify relevant articles from 2012 to March 2023. In addition, the reference list of potentially eligible studies was also screened to verify all relevant articles that may not have been identified during the database searches.

This systematic review wasl conducted following the Preferred Reporting Items for Systematic reviews and Meta-analysis (PRISMA) guidelines and recorded in Jan 25 2023 the OSF database with the Registration DOI: https://osf.io/9k8fw/.

The search strategies were based on the PICO(S) question “Will new diagnosis tools have the potential to change white spot’s conventional treatment?”, developed for the Pubmed database and adapted for Scopus database. The results of the different bases were crossed to locate and eliminate the duplications.

Below is the defined PICO (S) question:

(Patient/Problem): In patients with white spot lesion, I (intervention): new diagnosis tools, C (comparison): comparison between differential diagnostic tools and conventional ones, O (outcome): the treatment, S (study type): systematic review.

The complete search strategy for PubMed is shown:

(“White spot lesions” OR “White spots” AND (dental caries OR caries) AND (diagnose.AND treatment).

For the Scopus database, the following strategy was used:

TITLE-ABS-KEY== (“White spot lesions” OR “White spots” AND (dental caries OR. caries) AND (diagnosis AND treatment).

This systematic review included randomized controlled trials (RCT), cross- sectional and longitudinal studies complying the following inclusion criteria: (i) being in humans, (ii) being in English, (iii) being about white spot lesions, (iiii4) have been published between 2012 and 2023, (iiiii) having both diagnosis and treatment and (iiiiii) having full text available. In this review we excluded other systematic reviews of clinical trials and studies in vitro.

To be able to discuss and analyze the results obtained and to answer the PICO question, were created two groups (visual and clinical photographs vs fluorescence, Diagnodent, microcomputed tomography and microradiography) considering that the visual and clinical photographs group corresponds to the conventional method and the other group to the new diagnostic techniques.

After the literature search, two independent researchers (PL and TC) filter relevant articles that fit the study, analyzing the title and abstract for study selection. Any disagreements between reviewers was discussed with a third author (ACVMM). Cohen’s Kappa test was performed to assess reviewers’ agreement. Rayyan’s Intelligent Systematic Review Platform was used to assist in the systematic review process [15].

Reviewers extracted data independently from the articles selected for analysis. The information collected during data collection was as follows: title of the article, year of publication, authors, study design, type of participants, number of participants, age of participants, type of diagnosis and treatment, period of clinical follow-up and the evaluated outcome.

Revised Cochrane risk of bias tool for randomized trials (RoB 2.0 [16] This analysis is based on five domains: D1 (Bias arising from the randomized process), D2 (Bias due to deviations from intended intervention), D3 (Bias due to missing outcome data), D4 (Bias in measurement of the outcome) and D5 (Bias in selection of the reported result). In each article, the five domains were evaluated and, in each domain, one of the following judgments were applied: low risk, some concerns and high risk. In the end, the most prevalent judgment was the one that determined the final evaluation of each article. To evaluate methodological quality of cross-sectional, longitudinal studies and case reports, the critical assessment tool - Joanna Briggs Institute (JBI) was used [17].

## Results

In February 2023 data collection was performed and a systematic literature search identified 143 references potentially relevant, with 99 publications from the PubMed/Medline database, 33 from Scopus and 11 from manual search to consider grey literature. Two duplicates were found and excluded. Based on the information provided in the title and abstract, 90 articles were considered ineligible. The main reasons for non-inclusion were: (1) being a systematic review, (2) being in vitro and (3) not being about white spot lesions. Fifty-one articles were analyzed in full to collect more detailed information. Twenty-nine studies were excluded for the following reasons: (1) not having full text available and (2) not having diagnosis and treatment. Finally, after applying quality assessment tools (k = 0.91), 20 studies were included in the following review, with publication dates between 2012 and 2021 were included, 3 of which were published in 2012, 4 in 2013, 2 in 2014, 2 in 2016, 1 in 2017, 3 in 2018, 4 in 2020 and 1 in 2021. The different studies were carried out in different and varied countries such as the United States, Switzerland, Turkey, Norway, Egypt and India. The number of individuals in each study varied from just 5 to 115 people. The ages of the participants varied, from children aged 8 to adults aged 27. Intervention times were also not consensual and varied between a week and 18 months.

**Fig. 1:**
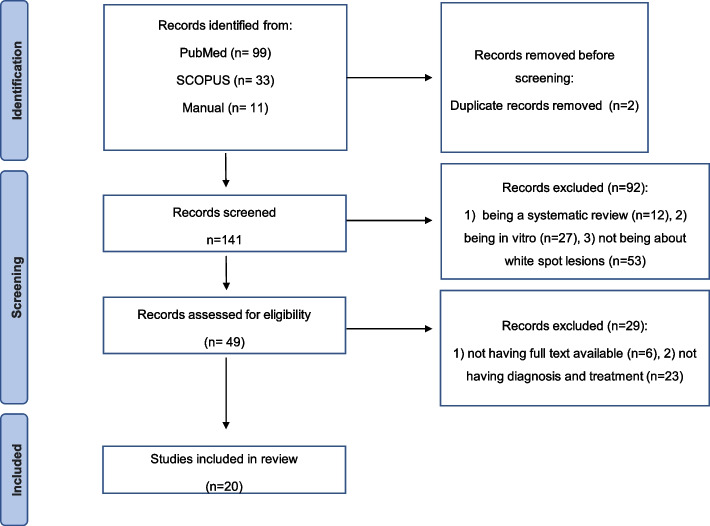
Overview of article selection procedure according to PRISMA guidelines

The study selection process is shown in Fig. 1.

Table 1, shows the primary characteristics of the 20 studies selected for this systematic review. Most of the studies selected are RCT [18–20, 22, 23, 25–27, 29, 35–37]. There are also cross-sectional studies [21, 28, 30, 32, 33], longitudinal studies [24, 34] and only one case report [31]. Visual and fluorescence are the most frequent diagnostic techniques, but other techniques such as clinical photographs [18, 19] and DIAGNOdent [35–37] are also used. Interestingly, one studie reported the use of two techniques: visual and fluorescence and fluorescence and transverse microradiography [29].

**Table 1:** Characteristics of the 20 studies selected for this review

Author	Study design	Type of participants	Nr of subjects	Age of participants	Intervention time	Diagnostic	Treatment	Outcomes	Conclusions
Sedlakova Kondelova et al., 2020 [18]	RCT	Patients presenting 2 teeth with WSLs	44	27	3 months	Clinical photographs	SAP P11-4	SAP P11-4 lesions showed significant WSL size reduction compared to FV alone	Treatment of early buccal carious lesions with SAP P11-4 led to superior regression of caries decay compared to either placebo or FV
Senestraro et al., 2013 [19]	RCT	Orthodontic patients	No information	No information	8 weeks	Clinical photographs	RI	The results for treated teeth showed a mean reduction in WSL area of 61.8% immediately after treatment and 60.9% eight weeks later	Resin infiltration significantly improved the clinical appearance of WSLs
Ciftci et al., 2018 [20]	RCT	No information	No information	No information	3 months	Visual	RI, FV	A significant decrease in DIAGNOdent Pen scores was observed in all the groups	The RI application was more successful than FV on WSLs
Hadler-Olsen et al., 2012 [21]	Cross-sectional	Orthodontic patients	80	12-16	18 months	Visual	Home care	23% of treated patients showed good compliance, 68% moderate compliance, and 9% poor compliance.	Individuals with good adherence developed fewer new WSLs than individuals with poor adherence
Kirschneck et al., 2016 [22]	RCT	Adolescent orthodontic patients	90	No information	20 weeks	Visual	Elmex® fluid and Fluor Protector S	Each treatment group showed a significant increase of the ICDAS index	A one-time application of FV at the start of orthodontic treatment did not provide any additional preventive advantage
Huang, et al. 2013 [23]	RCT	Orthodontic patients	115	12-20	8 weeks	Visual	MI paste plus, FV	The objective improvements in the affected surface were 16, 25 and 17% in the MI Paste Plus, fluoride varnish and control groups, respectively	MI Paste Plus and FV do not appear to be more effective than normal home care for improving the appearance of white spot lesions over an 8-week period.
Hammad et al., 2012 [24]	Longitudinal	Orthodontic patients	18	No information	No information	Visual	RI	Results after Icon application showed that around 65-76% of the surface area of the WSLs was masked	The masking effect depends on lesions depths
Krithikadatta et al., 2013 [25]	RCT	Patients with occlusal WSLs	45	No information	30 days	Visual	CPP-ACP, CCP-ACP with fluoride, NaF mouthwash	All three remineralising agents heal WSL	All three remineralising agents heal WSL.
Bock et al., 2017 [26]	RCT	Orthodontic patients	39	No information	24 weeks	Visual	Fluoride gel	No statistically significant group difference existed	No significant positive effect of high-dose fluoride on post-orthodontic WSL development could be detected
Rechmann et al., 2018 [27]	RCT	Orthodontic patients	37	No information	12 months	Visual	MI paste plus, MI varnish	Salivary fluoride levels were significantly higher at 12 months for the experimental than for the control group	Applying daily MIPP and quarterly MIV resulted in no statistically significant differences in ICDAS
Roig-Vanaclocha et al., 2020 [28]	Cross- sectional	No information	No information	No information	No information	Visual	HCL	When each application was evaluated with the initial situation of the untreated tooth, we observed that 6.6% HCl removes more enamel than 15% HCl	Both HCl-based products are adequate options for treating white spot lesions
Giray et al., 2018 [29]	GRCT	WSLs on permanent teeth in children	23	8-14	6 months	Visual and fluorescence	RI, FV	The values of the RV group were statistically lower than those of the FV group	RI and FV are clinically feasible and efficacious methods for the treatment of anterior WSLs
Marouane et al., 2021 [30]	Cross- sectional	Patients with MIH lesions on permanent anterior teeth	No information	No information	No information	Fluorescence	RI	A non-linear correlation was observed indicating that the IPx was rapid at the beginning of resin application, decreasing over time	MIH-lesion type and the ‘ethanol test’ were reliable predictive factors for the application time needed for infiltrating MIH lesions on permanent anterior teeth
Marouane et al., 2020 [31]	Case report	Patients with enamel opacities related to MIH	No information	No information	1 week	Fluorescence	RI	The lesions showed a clearly improved esthetic integration and had almost disappeared	Transillumination was also reliable in monitoring the progression of the infiltration until complete saturation of the porous enamel
Sezici et al., 2020 [32]	Cross- sectional	Orthodontic patients	57	No information	No information	Fluorescence	RI	Fluorescence of WSLs improved significantly following infiltration treatment in both groups	Immediate infiltration of clinically diagnosed WSLs can be the primary choice of treatment in orthodontic patients with poor oral hygiene and low remineralization expectancy
Tassery et al., 2013 [33]	Cross- sectional	Patients with carie	No information	No information	No information	Fluorescence	RI	The objectives of preserving the natural tooth structure are achieved	Combining the ultraconservative, restorative approach with a substantial caries risk assessment and caries management with remineralization programme may provide therapeutic benefits
Kabaktchieva et al., 2014 [34]	Longitudinal	Patients with carie	No information	No information	1 year	Fluorescence	RI	The lesion progression could be detected very precisely. When the demineralization is slight Icon fills all the pores in the body of the lesion and the camera detects sound tissues	LIF method applied with SoproLife camera is more accurate than visual examination. LIF method for single tooth is more accurate in following up the effect of non-operative treatment of smooth surfaces lesions than using digital images. ICON is a material that stops the progression of non-cavitated smooth surfaces carious lesions and make the aesthetic result better
Perrini et al., 2016 [35]	RCT	Orthodontic patients	24	No information	No information	DIAGNOdent	FV	The varnished anterior teeth showed a statistically significant reduction in demineralization compared with their unvarnished counterparts	Periodic application of FV can offer some protection against WSLs, but not to a statistically significant degree if the patients have excellent oral hygiene
Du et al., 2012 [36]	RCT	Orthodontic patients	110	12-22	6 months	DIAGNOdent	FV	There was statistically significant differences between the mean DD readings of the two groups at the 3-month (*P* < 0.05) and at the 6-month follow-up visits (*P* < 0.01).	Topical FV application is effective in reversing WSLs after debonding and should be advocated as a routine caries prevention measure after orthodontic treatment
Aykut-Yetkiner et al., 2014 [37]	RCT	Children exhibiting at least 1 WSL	60	No information	3 months	DIAGNOdent	CPP-ACP	In both groups, the mutans counts were decreased	CPP-ACP had a slight remineralization effect on the WSL

Most of the studies reported the use of infiltrating resin and fluoride varnish as treatment techniques [19, 20, 23, 24, 29–36].

However, the treatment with self-assembling peptide P11-4 (SAPP11-4) [18], home care [21], Elmex® fluid [22], CPP-ACP [25, 37], fluoride gel [26], MI paste plus and MI varnish [27] and Hydrochloric acid HCL [28])were taken in to account.

In what concerns the intervention time, it is notorious the discrepancy between the studies. The study with lower intervention time reported the use of treatment techniques for 1 week [31]. However, more robust studies within 3, 5, 6, 9, 12 or 18 months of using the treatment techniques [18, 20–22, 26–28, 34, 36, 37] are most of the cases.

The impact of treatment considering diagnostic techniques was evaluated in a range of different cohorts, as children, adolescents, and adults all in good state of general systemic health. The studied population has several characteristics related to orthodontic treatment [19, 21–24, 27, 32, 35, 36]MIH [30, 31] and carie [18, 33, 34]. The caries identification and evaluation method were mostly accessed by the International Caries Detection and Assessment System (ICDAS II), however other clinical evaluation criteria were also considered.

In the various studies included, different ways of carrying out the diagnosis and treatment were used. Thus, most studies showed positive results, in which the treatment methods used improved or even reversed the white spot lesion on tooth surfaces. Only one study did not show significant statistical differences in treatment with the use of fluoride gel [26].

The use of SAPP11-4 was found to be successful compared to the use of fluoride varnish alone in regressing dental caries [18].

In all studies carried out with infiltrating resin, it presented good clinical results in the aesthetic improvement of the white spot lesion and tooth remineralization [19, 30–34]. Compared to the use of fluoride varnish, it may even provide better evidence than fluoride varnish [20], although a study mentioned the opposite [29]. Some studies report that lesion improvement with infiltrating resin is related to its depth [24].

Regarding the use of fluoride varnish, the study that resorted only to this form of treatment show good results, stating that there is a positive change at the mineral level and that it is a good way to prevent the existence of dental caries [35]. Although the difference is not very significant when applied to patients with good oral hygiene [36]. This is also reported in another study which refers that the use of fluoride varnish compared to home care does not present major differences in the treatment of white spot lesions [23]. Likewise, it is reported that an application of fluoride varnish before orthodontic treatment as prevention is not relevant [22]. Fluoride-based products and home care have proven to be helpful in treating injuries, but compliance by the patients themselves also must be good [21, 27].

The use of CPP-ACP had good remineralization effect in white spot lesions [25, 37].

The use of HCL also showed good results for the treatment of white spot lesions, despite the percentage of HCL being related to the amount of enamel removal [29].

**Fig. 2:**
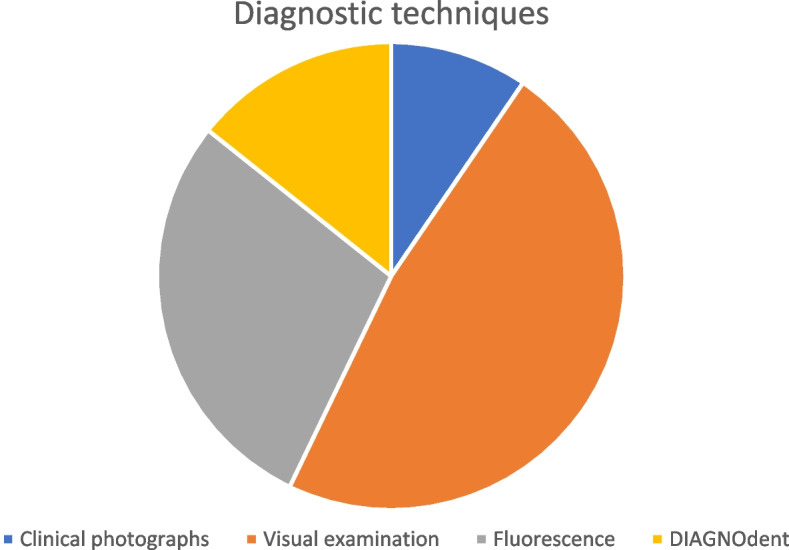
Diagnostic techniques included in this systematic review

**Fig. 3:**
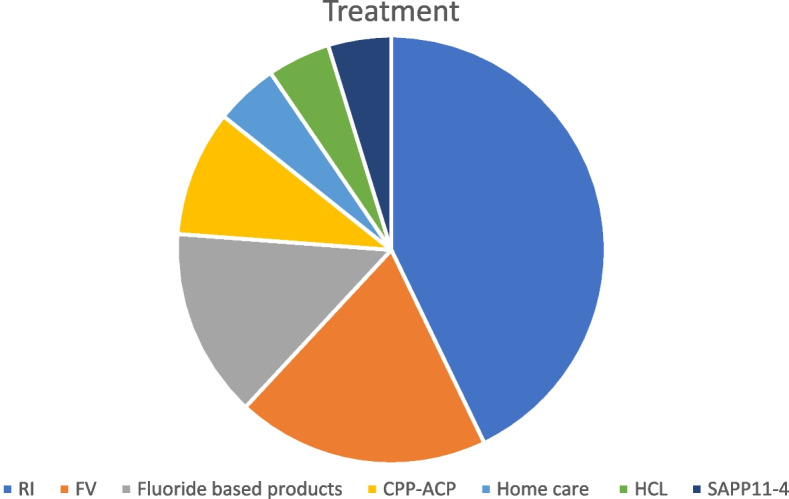
Assessment treatment techniques included in this systematic review

In the following figures, Figs. 2 and 3, it is possible to observe, through two different graphics, the number of studies that used the different diagnostic methods (Fig. 2) and treatments (Fig. 3) in this systematic review. So that in this way, you can have a better perception of the number of studies that refer to each type of method of diagnosis and treatment.

Regarding diagnostic methods, 2 of the 20 used clinical photographs as a diagnostic method, 9 used visual examination, 5 used fluorescence, 1 used microcomputed tomography and 3 used DIAGNOdent. A combination of two methods, visual examination and fluorescence, was also used.l.

Regarding treatment methods, 7 out of 20 used infiltrating resin, 5 used fluoride-based products, 1 used SAPP11-4, 1 used home care, 2 used CPP-ACP and 1 used HCL. Combination therapies were also considered: 2 used fluoride varnish and 2 used infiltrating resin and fluoride varnish.

### Risk of bias analysis

Table 2 shows the risk of bias analysis using the RoB tool. This tool consists of evaluating articles in five different domains.

**Table 2:**
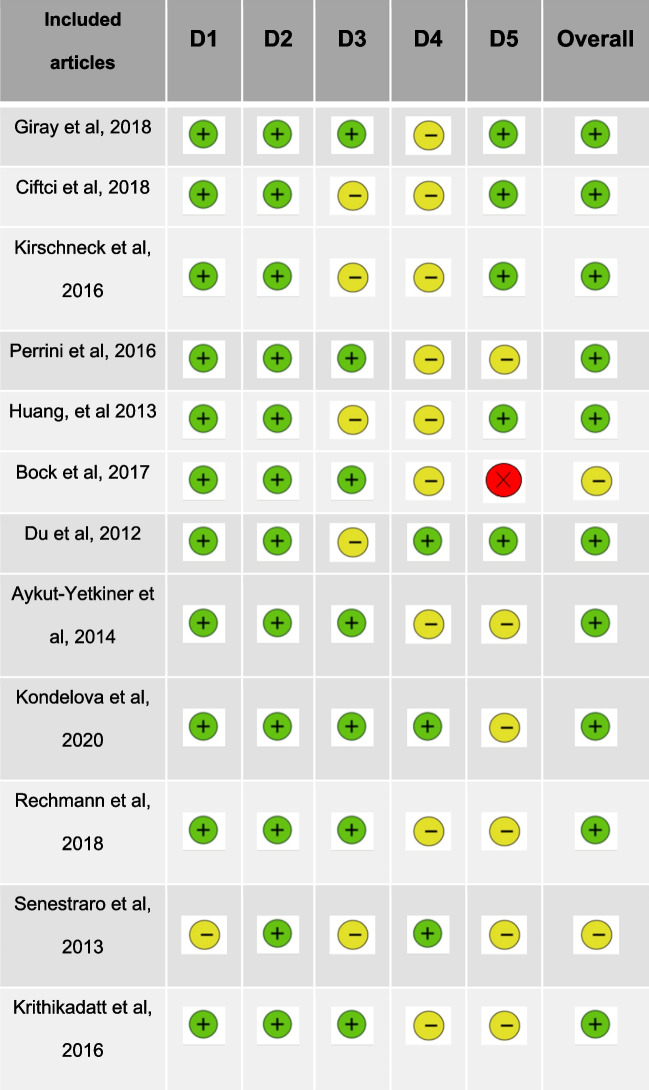
Risk of bias analysis according to the RoB tool to RCT studies (Cochrane Library) [18–20, 22, 23, 26, 27, 29, 35–37]

The first domain evaluates Bias arising from the randomized process. In this domain, 11 articles have a low risk of bias and 1 article has some concerns.. Domain two refers to Bias due to deviations from intended intervention. In this domain, all articles have a low risk of bias. Regarding the third, Bias due to missing outcome data, 7 articles have low risk and 5 article hase some concerns.. Domain four is about Bias in measurement of the outcome. In this domain, 9 articles present some concerns and3 low risk. In the last domain, which addresses Bias in selection of the reported result, 5 articles present low risk, 6 present some concerns and only 1 presents high risk. Overall, this analysis resulted in 10 of the 20 articles with low risk of bias and the remaining 2 articles with some problems.

Cross-sectional studies, longitudinal and case reports were analyzed regarding the quality of the study according to the JBI criteria and the results of the analysis are presented in Tables 3, 4, and 5. Almost all aspects of the analysis were fulfilled except with regard to confounding factors. In most articles this aspect was not identified and strategies to deal with confounding factors were not always stated.

**Table 3:** Risk of bias according to JBI, the critical assessment tool, to cross sectional studies

Author	1. Were the criteria for inclusion in the sample clearly defined?	2. Were the study subjects and the setting described in detail?	3. Was the exposure measured in a valid and reliable way?	4. Were objective, standard criteria used for measurement of the condition?	5. Were confounding factors identified?	6. Were strategies to deal with confounding factors stated?	7. Were the outcomes measured in a valid and reliable way?	8. Was appropriate statistical analysis used?
Roig-Vanaclocha et al., 2020 [28]	**Yes**	**No**	**Yes**	**Yes**	**In part**	**In part**	**Yes**	**Yes**
Marouane et al., 2021 [30]	**In part**	**No**	**Yes**	**Yes**	**In part**	**In part**	**Yes**	**Yes**
Sezici et al., 2020 [32]	**Yes**	**Yes**	**Yes**	**Yes**	**In part**	**In part**	**Yes**	**Yes**
Tassery et al., 2013 [33]	**Yes**	**No**	**Yes**	**Yes**	**In part**	**No**	**Yes**	**Yes**

**Table 4:** Risk of bias according to JBI, the critical assessment tool, to longitudinal studies

Author	1. Were the criteria for inclusion in the sample clearly defined?	2. Were the study subjects and the setting described in detail?	3.Was the exposure measured in a valid and reliable way?	4.Were objective, standard criteria used for measurement of the condition?	5.Were confounding factors identified?	6.Were strategies to deal with confounding factors stated?	7.Were the outcomes measured in a valid and reliable way?	8.Was appropriate statistical analysis used?
Kabaktchieva et al., 2014 [34]	**Yes**	**Yes**	**Yes**	**Yes**	**In part**	**In part**	**Yes**	**In part**
Hammad et al., 2012 [24]	**Yes**	**Yes**	**Yes**	**Yes**	**In part**	**In part**	**Yes**	**Yes**

**Table 5:** Risk of bias according to JBI, the critical assessment tool, to case reports

Author	Were patient’s demographic characteristics clearly described?	Was the patient’s history clearly described and presented as a timeline?	Was the current clinical condition of the patient on presentation clearly described	Were diagnostic tests or assessment methods and the results clearly described?	Was the intervention(s) or treatment procedure(s) clearly described?	Was the post-intervention clinical condition clearly described?	Were adverse events (harms) or unanticipated events identified and described?	Does the case report provide takeaway lessons?
Marouane et al., 2020 [31]	**In part**	**In part**	**Yes**	**Yes**	**Yes**	**In part**	**In part**	**Yes**

## Discussion

It is known that dental caries is a very common pathology, of bacterial origin and that can be noticed, at an early stage, in the form of a white spot lesion. It is known that it is the most common oral pathology, which can affect all individuals of different ages and from different populations, particularly the most socioeconomically vulnerable [17].

Thus, since the high worldwide prevalence of this pathology, this study is very important and relevant. In an attempt to reduce its existence, it is important to study and know both the diagnosis and treatment of caries and, particularly, of the white spot lesion. In this way, we will be able to timely identify and therefore act against this pathology still in an early stage. As well as knowing how to act in the intervention area in order to reduce the prevalence rate of caries and white spot lesions.

In this way, a large number of clinical studies was obtained after searching the various databases [18, 20, 22, 24, 28, 29, 31–35, 38]. This shows the usefulness and relevance of carrying out a systematic review of this subject. Also, because there are variations in the types of white spot lesions and, therefore, it is important to understand the longevity of the appropriate treatment for each clinical situation.

Thus, to allow a better discussion of this topic, the study was based on a PICO question and the PRISMA model. The Rayyan and RoB tools were also used in order to help visualize and operationalize the selection and quality of articles since they are both well accepted in systematic reviews. With this, it was verified that most of the studies were of good quality.

As said before, to discuss and analyze the results obtained and to answer the PICO question, were created two groups (visual and clinical photographs vs fluorescence, Diagnodent, microcomputed tomography and microradiography) considering that the visual and clinical photographs group corresponds to the conventional method and the other group to the new diagnostic techniques.

The choice of diagnostic technique to be used for detecting caries and white spot lesions is important and depends on the clinical case. It is important to understand whether these tools have enough scientific evidence to be chosen over conventional diagnostic techniques and that allow the treatment method to be also differentiated and less invasive than the current one.

Several treatments were reported in studies in which conventional diagnoses such as: SAPP11-4, infiltrating resin, fluoride varnish, hygiene care at home, CPP-ACP, HCL and even chlorhexidine varnish. Therefore, there was no consensus in all articles concerning the treatment associated with conventional diagnostic techniques, although the results were satisfactory in all studies.

The result considered good and satisfactory may have a subjective character since the parameters for evaluating the results are not the same for all articles, which means that the results cannot be evaluated and perceived in exactly the same way.

On the other hand, the treatments reported in the studies in which the diagnoses were differentiated consisted of infiltrative resin, fluor varnish, hygiene care at home with fluoride products andCPP-ACP.

The choice of treatments applied after using different diagnostic techniques was also not consensual, as there was variation in the choice of treatment and the results were also all positive. Thus, all means of treatment promote good evidence in the teeth of patients.

Resin is a more immediate means of treatment that improves esthetics, leads to short- and long-term improvements in tooth decay and white lesions, and does not limit other dental treatments.

Fluoride, whether applied in the form of varnish or in the form of other fluoride products, helps prevent caries and can reverse initial lesions, strengthening the enamel structure and reducing tooth sensitivity. Unlike resin, this mineral product ends up being a less immediate means of treatment, as results are not observed right after its application.

CPP-ACP products also have advantages such as enamel remineralization, the ability to treat and prevent the occurrence of white spots. These products are easy to apply and help with tooth sensitivity, being compatible with the use of fluoride.

The use of HCL in the treatment of white spot lesions is related to its ability to decontaminate and demineralize the injured areas so that the consequent restorative treatment is possible and more effective.

SAPP11-4 is also a product that can be used in combination with fluorine, not limiting its action and promoting enamel remineralization. This monomeric self-assembling peptide solution can diffuse into carious lesions and promote hydroxyapatite formation, significantly reducing the size of buccal white spot lesions [28].

All these products must be used with expertise and training as they can cause other injuries if misused, as is the case with HCL.

According to several studies, both infiltrating resin and the application of fluoride varnishes are effective in the treatment of white spot lesions. The result of treatment with resin is immediate, while fluoride must be applied for a certain period of time for its effect to be noticed. If the continuous release of fluoride manages to combat enamel demineralization, its application will be enough to treat the lesion. Therefore, intervention with infiltrating resin is not necessary. This makes the use of fluoride a non-invasive method. Some studies also consider infiltrating resin to be a less invasive method with many advantages. One of the great advantages refers to the ICON material, which can improve the aesthetics of deciduous and permanent teeth. Thus, immediate resin treatment may be a good option in patients whose level of hygiene is poor, with little likelihood of remineralization [20, 29, 32, 34].

In this way, the application of fluoride varnish has more benefits in uncooperative patients and with a greater lack of hygiene. That is, in patients with good oral hygiene and a low risk of dental caries, the application of fluoride varnish may not show noticeable improvements compared to home hygiene care [22, 35].

It was also found that CPP-ACP products combined or not with fluoride are effective in the treatment of white spots, being better than just mouthwashes with fluoride products. Although fluoride products are also effective for tooth remineralization [18, 29].

Studies demonstrate that SAPP11-4 is effective for treating lesions. Since the product does not inhibit the action of fluorine, it can even be applied together with fluorine. Especially because SAPP11-4 ends up having better results than fluor individually. This is a minimally invasive and innovative technique capable of inducing the formation of hydroxyapatite in the lesion itself [28].

Regarding the use of HCL, it is known to be useful in the treatment of white spot lesions. Thus, the application of acid prior to restoration with infiltrative resin allows the surface layer of the lesion to weaken and shrink. However, the use of this product causes corrosion of the enamel surface and, therefore, its application must be careful so that, subsequently, the adhesion and the performance of the restoration are not compromised. Just as care is needed not to compromise the gum, leading to the occurrence of ulcers [18, 24].

Regarding the diagnosis, it is essential that the dentist does not lose the ability to detect a carious lesion or a white spot through visual or photographic examination, as is the most conventional way. However, other forms of diagnosis have emerged, such as fluorescence and DIAGNOdent, as well as microradiography and microcomputed tomography to facilitate and accelerate the diagnostic process.

Fluorescence is useful because it allows more precise and intuitive detection of lesions since it is done through the emission of light. Then, the DIAGNOdent appears, which is a device that uses fluorescence and is minimally invasive and painless, using a quantitative method to assess the severity of the lesion. Fluorescence should not be used individually, but as an aid to the visual diagnosis made by the dentist.

Transverse microradiography is a diagnostic technique used, comparing photographs at different points, also to quantitatively assess the mineral content and perceive the state of remineralization/demineralization of the teeth. Regarding diagnostic methods, in general, lesion values decreased, and lesions improved when DIAGNOdent was used [20].

Fluorescence was useful in detecting demineralization and remineralization of lesions. This method has been shown, in several studies, to be favorable in assessing the size of the lesion and in evaluating its progression throughout the treatment. The use of fluorescence is quite accurate and ends up surpassing the visual examination. However, it is important to maintain visual inspection skills so that the clinician does not lose his diagnostic sensitivity when inspecting and detecting a lesion [31, 33, 34].

All studies were carried out on individuals of different ages and from different socioeconomic backgrounds, with some reporting the use of orthodontic appliances. In this way, we can observe that the results will be more comprehensive and real.

In general, the studies addressed less invasive techniques, as they considered that acceptance by patients would be better and that the treatment itself would also have advantages the more conservative it was.

Hence, it is not possible to reach a rigorous conclusion. Therefore, in order to draw more conclusions from this study, it would be convenient to follow up on the cases in order to see if the treatments carried out with the differentiated methods have better durability than the treatments carried out with conventional diagnostic methods.

Thus, several conflicting results regarding the type of diagnosis and treatment applied to white spot lesions exist. Although all forms of treatment improve the lesion, some are more invasive than others.

Therefore, both through a conventional diagnosis and through a differentiated diagnosis, the form of treatment does not present great differences and may be the same. This means that there is no scientific evidence of less invasive treatment techniques than the current ones in the face of a differentiated diagnosis. Thereby, the chosen diagnosis type will not change the clinical approach to a white spot lesion.

It’s important to remind that, as the main motivation of patients with white spot lesions is fundamentally aesthetic, follow-up is very important. In terms of treatment, the patient values the result and especially the stability of the treatment carried out, and the professional must be able to guarantee this to patients.

## Conclusion

There are several options for the diagnosis, as well as for the appropriate treatment of white spot lesions, with a positive prognosis and good treatment longevity.

With this systematic review, it is possible to conclude that the diagnostic tool chosen does not have the potential to change the treatment option, whether it is a conventional tool or a different one.

Therefore, there are no differences in the therapeutic approach for the treatment of white spot lesions, regardless of the type of diagnosis used.

## Data Availability

All data generated or analysed during this study are included in this published article.

## Abbreviations

WSL: White Spot Lesion
MIH: Molar-incisor hypomineralization
ICDAS: International Caries Detection and Assessment System
CPP-ACP: Casein phosphopeptide-Amorphous calcium phosphate casein
SAPP11-4: Self-assembling peptide P11-4
IR: Resin Infiltration
FV: Fluoride Varnish
HCL: Hydrochloric acid
MI: Minimally invasive
NaF: Sodium fluoride
RCT: Randomized clinical trial
MIV: Minimally invasive varnish
MIPP: Minimally invasive paste plus
IPx: Infiltration proportion
DD: DIAGNOdent
LIF: Light-Induced Fluorescence
QLF- D: Quantitative Light-induced Fluorescence: Digital System
RCT: Randomized clinical trial
TMR: Transverse microradiography
DDE index: Enamel Developmental Defects index
